# Association of Genetic Variants of Melatonin Receptor 1B with Gestational Plasma Glucose Level and Risk of Glucose Intolerance in Pregnant Chinese Women

**DOI:** 10.1371/journal.pone.0040113

**Published:** 2012-07-02

**Authors:** Shunyao Liao, Yunqiang Liu, Yuande Tan, Lu Gan, Jie Mei, Wenzhong Song, Shu Chi, Xianjue Dong, Xiaojuan Chen, Shaoping Deng

**Affiliations:** 1 Diabetes Center, Sichuan Academy of Medical Science and Sichuan Provincial People's Hospital, Chengdu, China; 2 Department of Medical Genetics and Division of Morbid Genomics, State Key Laboratory of Biotherapy, West China Hospital, Sichuan University, Chengdu, China; 3 College of Life Science, Hunan Normal University, Changsha, Hunan, China; 4 Department of Obstetrics and Gynecology, Sichuan Academy of Medical Science, Sichuan Provincial People's Hospital, Chengdu, China; 5 Clinical Isotopic Laboratory, Sichuan Academy of Medical Science, Sichuan Provincial People's Hospital, Chengdu, China; 6 Department of Endocrinology, Chongqing Medical University, Chongqing, China; 7 Department of Surgery, Northwest University Hospital, Chicago, Illinois, United States of America; 8 Human Islet Laboratory, Massachusetts General Hospital, Harvard Medical School, Boston, Massachusetts, United States of America; Virgen Macarena University Hospital, School of Medicine, Spain

## Abstract

**Background:**

This study aimed to explore the association of *MTNR1B* genetic variants with gestational plasma glucose homeostasis in pregnant Chinese women.

**Methods:**

A total of 1,985 pregnant Han Chinese women were recruited and evaluated for gestational glucose tolerance status with a two-step approach. The four *MTNR1B* variants rs10830963, rs1387153, rs1447352, and rs2166706 which had been reported to associate with glucose levels in general non-pregnant populations, were genotyped in these women. Using an additive model adjusted for age and body mass index (BMI), association of these variants with gestational fasting and postprandial plasma glucose (FPG and PPG) levels were analyzed by multiple linear regression; relative risk of developing gestational glucose intolerance was calculated by logistic regression. Hardy-Weinberg Equilibrium was tested by Chi-square and linkage disequilibrium (LD) between these variants was estimated by measures of D′ and r^2^.

**Results:**

In the pregnant Chinese women, the *MTNR1B* variant rs10830963, rs1387153, rs2166706 and rs1447352 were shown to be associated with the increased 1 hour PPG level (*p* = 8.04×10^−10^, 5.49×10^−6^, 1.89×10^−5^ and 0.02, respectively). The alleles were also shown to be associated with gestational glucose intolerance with odds ratios (OR) of 1.64 (*p* = 8.03×10^−11^), 1.43 (*p* = 1.94×10^−6^), 1.38 (*p* = 1.63×10^−5^) and 1.24 (*p* = 0.007), respectively. *MTNR1B* rs1387153, rs2166706 were shown to be associated with gestational FPG levels (*p* = 0.04). Our data also suggested that, the LD pattern of these variants in the studied women conformed to that in the general populations: rs1387153 and rs2166706 were in high LD, they linked moderately with rs10830963, but might not linked with rs1447352;rs10830963 might not link with rs1447352, either. In addition, the *MTNR1B* variants were not found to be associated with any other traits tested.

**Conclusions:**

The MTNR1B is likely to be involved in the regulation of glucose homeostasis during pregnancy.

## Introduction

The number of women with gestational carbohydrate intolerance (abnormal oral glucose tolerance test results) including gestational diabetes mellitus (GDM, glucose intolerance of any degree with onset or first recognition during pregnancy) is increasing world-wide and the prevalence varies considerably among racial and ethnic groups [1]. Recent studies reported that adverse maternal-fetal outcomes were observed across the spectrum of carbohydrate intolerance including glucose values below current cutoffs for the diagnosis of GDM [2], [3]. Similarly, both GDM and mild gestational carbohydrate intolerance in women may lead to a high risk of metabolic syndromes related to cardiovascular disease, type 2 diabetes, breast cancer and dementia later on in life [3], [4]. Gestational glucose abnormalities are associated with perinatal complications and the subsequent development of metabolic diseases. The retrospective and prospective studies on gestational glucose metabolism, however, are complicated as the GDM and gestational glucose intolerance are transient phenomena resulting from transient rises in serum levels of the placenta hormones [5]. In addition, although several studies have examined the association of type 2 diabetes susceptibility loci with GDM [6], [7], [8], [9], there is insufficient genetic evidence for the gestational carbohydrate intolerance; and so far, little knowledge is available regarding the predisposition of gestational carbohydrate homeostasis in pregnant Chinese women.

Melatonin is a circulating hormone from the pineal gland and regulates seasonal and circadian rhythms. MTNR1B belongs to one of the functional melatonin receptor subtypes [10] and is expressed in pancreatic islets [11]. The Genome-wide Association Studies (GWAS) have provided strong supports for the associations of genetic variations in the *MTNR1B* locus with FPG, glucose level, insulin secretion and type 2 diabetes [11]–[19]. Up to now, four *MNTR1B* genetic variants have been reported to be associated with phenotypes: rs10830963 has been shown to influence FPG in people of European descent [11], [12], [13] and to be associated with type 2 diabetes in Han Chinese individuals [14]; rs1387153 has been reported to be associated with increased FPG and risks of type 2 diabetes [15], [16], [17]; rs2166706 has been demonstrated to cause rises in plasma glucose levels as well as in the risk of type 2 diabetes in European Caucasians and Indian Asians [18]; and rs1447352 has been found to be associated with glucose metabolism [19]. The interrelationships between these variants are available in HapMap database (http://hapmap.ncbi.nih.gov). These variants are shown to be in moderate to strong LD and the relation between LD blocks is somewhat different among general Europeans and Chinese populations [20]. In short, the GWAS indicate that the ‘clock’ gene may be involved in glucose metabolism and its regulation may relate to the pathogenesis of type 2 diabetes [21]. Since glucose intolerance in the general population and in pregnant women share a common pathophysiological background and women with glucose intolerance during pregnancy have a higher prevalence of type 2 diabetes after pregnancy, we hypothesize that the *MTNR1B* genetic predisposition of gestational glucose metabolism is similar to that of the general non-pregnant population. This study was designed to address the possible associations between the *MTNR1B* genetic variants and the gestational glucose homeostasis in a pregnant Han Chinese population.

## Materials and Methods

### Study population

The study recruited 1,985 pregnant Chinese women who received prenatal care in 17 different Hospitals of Chengdu City in Southwest China (93.3% of all pregnant women from April 1, 2010 to April 1, 2011). All participants were Han Chinese and resided in the metropolitan area of Chengdu. A two-step approach based on National Diabetes Data Group (NDDG) diabetes criteria for diagnosis of gestational abnormal glucose tolerance and GDM was employed [22, Flowchart S1]. First, the pregnant women were routinely offered a 50 g, 1 hour (1 h) oral glucose tolerance test at 24–28 gestational weeks for the screening glucose challenge test(GCT), both levels of FPG and 1 h PPG were determined; Secondly, women with FPG ≥5.8 mmol/l or 1 h PPG ≥7.8 mmol/l in GCT and high-risk pregnant women [23] might also be offered a 100 g, 3 h oral glucose tolerance test (OGTT); those that had two or more abnormal values on OGTT, would be diagnosed with GDM; and those had one value over the threshold, would be considered to have gestational impaired glucose tolerance (GIGT). High-risk pregnant women with FPG ≥7.0 mmol/l or a random PPG ≥11.1 mmol/l that were confirmed on a subsequent day would be diagnosed with GDM. In this study, among different degree of abnormal glucose tolerance, ABN was defined as abnormal 50 g GCT or OGTT results except GIGT and GDM. Three women with type 1 diabetes and 4 women who had been previously diagnosed with type 2 diabetes were excluded.

The OGTT was analyzed by a chemiluminescent immunoassay using the ARCHITECT i2000SR System (Abbott) in the central Clinical Isotopic Laboratory of Sichuan Provincial Hospital. The glucose levels were measured to 0.11 mmol/l. The height and body weight of all the recruited pregnant women were measured to the nearest 0.1 cm and 0.1 kg, respectively. The BMI of each participant was calculated. The gestational age was calculated from the date of last menstrual period.

The clinical profiles of the 1,985 pregnant Chinese women are shown in Table 1. According to the NDDG criteria, 72 women were diagnosed with GDM (3.63%), 98 with GIGT (4.94%), 605 women with ABN (30.48%), and the remaining 1210 women with normal glucose tolerance (NGT, 60.96%). The averaged age, weight, BMI, and glucose levels are different between the NGT and the GIGT/ABN & GDM groups, indicating these 2 groups are distinct in clinical characteristics.

**Table 1:** Comparison of Characteristics in pregnant Chinese women.

	Normal glucose tolerance group	Abnormal glucose tolerance group				Difference of means between the normal and abnormal groups
Classified according to NDDG criteria	NGT(n = 1210)	GIGT(n = 98)	ABN(n = 605)	GDM(n = 72)	Total(n = 1985)	*p_*value
Age (year)	28.11±3.96	30.90±4.09	29.41±4.07	30.50±4.81	28.73±4.12	<10^−6^
Height (cm)	159.86±4.23	158.59±3.78	159.45±4.26	158.45±4.31	159.61±4.24	0.003
Gestational week at GCT (wk)	26.04±2.23	26.15±2.07	26.04±2.27	26.31±2.44	26.06±2.29	0.08
Weight (kg) at GCT	55.61±7.33	57.29±9.05	56.35±8.21	58.56±10.16	56.02±7.82	0.007
Weight (kg) before delivery	65.48±8.14	68.00±10.00	65.72±8.55	68.57±12.04	65.75±8.52	0.19
BMI (kg/m^2^) at GCT	21.76±2.77	22.77±3.39	22.17±3.03	23.11±3.77	21.98±2.94	0.0001
BMI (kg/m^2^) before delivery	25.55±3.06	26.71±3.64	25.86±3.12	27.56±4.20	25.75±3.17	0.007
Fasting plasma glucose level (mmol/l)	4.60±0.54	5.30±0.71	4.97±0.0.61	5.64±1.43	4.78±0.68	<10^−6^
postprandial 1 hour plasma glucose level (mmol/l)	6.36±0.85	9.40±1.03	8.76±0.94	10.28±1.83	7.38±1.61	<10^−6^

Data are presented as (mean ± SD).

### Ethical considerations

The written informed consent was obtained from each and every participant. The study protocol was approved by the Review Board of Clinical Research of the Sichuan Provincial hospital, Maternal and Child Health Hospital of Chengdu and by the Research & Ethics Committee of Sichuan Medical Research Institution.

### DNA extraction

Blood samples were collected as dried blood spots and total DNA was isolated from peripheral blood lymphocytes. 1,975 (99.5%) blood samples were collected from 50 g GCT and 10 (0.5%) were from 100 g OGTT. Briefly, erythrocyte lysis buffer was used to remove DNA-free erythrocytes, and nuclear lysis buffer to release DNA from lymphocytes. The protein precipitation solution was used to precipitate and remove protein selectively. Finally, the purified DNA was precipitated by isopropanol, resuspended in TE buffer and stored at −20°C for later genotyping. Gel electrophoresis and spectrophotometric determination were used to DNA quantification and quality analysis. DNA samples with OD260/OD280 ratio between 1.8–2.0 and concentration more than 50 ng/ml were used for genotyping.

### Genetic variants selection and Genotyping

**Figure 1:**
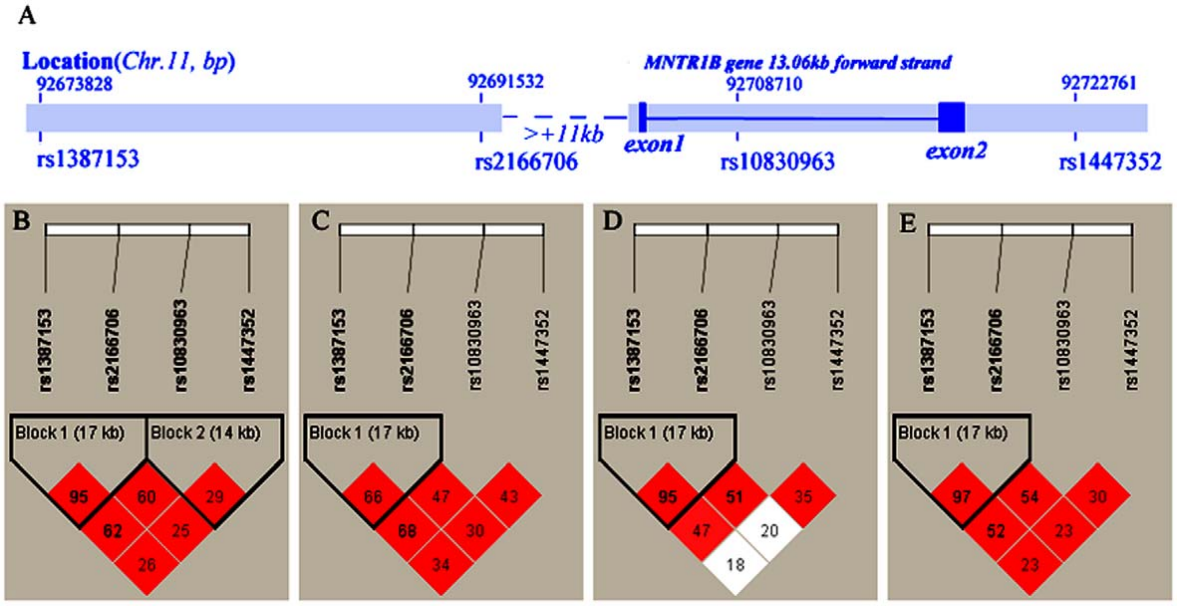
The *MTNR1B* gene structure and Haploview plot for 4 *MTNR1B* variants. The *MTNR1B* gene structure with the location of the variants studied (A) and pairwise D′ Haploview plot for the 4 *MTNR1B* variants was shown; R-squared was also displayed for LD values. B. based on our data for glucose tolerance status in pregnant Chinese women. C. Europeans. D. Han Chinese in Beijing. E. Chinese in Metropolitan Denver. 88×68 mm (600×600DPI).

We selected the 4 *MTNR1B* genetic variants rs10830963, rs1387153, rs2166706 and rs1447352 for genotyping since they had been reported to be associated with plasma glucose levels in general populations [11]–[19]. The gene structure of *MTNR1B* and the location of the 4 variants were shown in Figure 1A. The LD between these variants in HapMap database showed that all 4 variants were in moderate to high LD in general populations. The LD plots of the populations of Europeans, Han Chinese in Beijing and Chinese in Metropolitan Denver were shown in Figure 1C, D, E [20]. Though rs1387153 and rs2166706 were in the same LD block at |D′| = 1.00, r^2^ in Chinese (>0.95) were higher than that in Europeans (r^2^ = 0.67). On the other hand, although rs10830963 and rs1447352 seemed to be linked with high D′, they might not be located in the same LD block as the linkage was not perfect in all these populations (r^2^<0.45). Noticeably, rs10830963 was linked moderately with the LD block of rs1387153 and rs2166706 by both measures of D′ and r^2^, while rs1447352 was not linked with the LD block by measure of r^2^ among these populations. We estimated the LD between these variants based on our data in the pregnant Chinese women studied.

These 4 variants were genotyped with an ABI PRISM 3100 Genetic Analyzer and the sequenom system at the Huada Gene Laboratory (Shengzhen, China). The genotyping call success rate was >98%. Among the total 1,985 samples, 100 samples were run in duplicates with a >99% concordance rate (Quality control is shown in Table S1).

### Statistical Analysis

Pearson's Chi-square was conducted in pregnant Han Chinese women to test for Hardy-Weinberg equilibrium of each variant. The allelic and genotypic frequencies of all the 4 variants were in Hardy-Weinberg equilibrium (HWE *p*-values were showed in Table S1).

Statistical power for correlation and regression analysis was calculated by G*Power3.1 [24]. The levels of FPG and 1 h PPG from 50 g GCT were used for genetic analyses in the study. For FPG analysis, with the effect size of 0.068, 0.068, 0.07, and 0.065 mmol/l per allele under an additive genetic model according to previous researches in Europeans for rs10830963, rs1387153, rs2166706 and rs1447352, respectively [11], [18], [16], [19], our total sample size of 1,985 would have over 80% statistical power with a type I error rate of 0.05. Power calculation for PPG analysis, we used the effect size >0.1 mmol/l per allele based on our data. And statistical power of logistic regression was calculated by Power for Genetic Association software [25]. The prevalence of gestational glucose intolerance was calculated at 39%, with risk allele frequencies of 0.41, 0.58, 0.42 and 0.31 for rs10830963 G, rs2166706 T, rs1387153 T, and rs1447352 G allele according to previous researches in Han Chinese in Beijing, respectively, odds ration of 1.64, 1.40, 1.45 and 1.26, also for respective variants. Using these parameters, it was calculated that our sample size of 775 abnormal glucose level cases and 1,210 controls would have over 95% statistical power with a type I error rate of 0.05.

Linkage between these variants was calculated by the Haploview v4.1 software downloaded from website of the Broad Institute http://www.broadinstitute.org/mpg/haploview [26].

Under the additive model with adjustment of age and BMI, multivariate linear regression was performed to detect association between the polymorphisms and the gestational plasma glucose levels in all the pregnant Han Chinese women; to examine residual association of the outcome with the other variants, conditional analysis was carried out on the strongest signal in univariate analysis. Logistic regression analysis was utilized to evaluate the relative risk of gestational glucose intolerance of various degrees. In particular, since women with mildly elevated glucose levels also demonstrate a risk of metabolic syndromes and adverse outcomes, and an abnormal GCT in pregnancy, even followed with a normal OGTT, predicted risks of adverse obstetrical outcomes and metabolic dysfunction [27], we combined GIGT/ABN and GDM women into one abnormal group; thus, we divided the pregnant Chinese women into two groups: the normal group including all women with a regular result in any glucose tolerance tests and the abnormal group consisting of the women with various degrees of glucose intolerance including GIGT/ABN & GDM. These statistical analyses were performed using STATA software, 2011 release (StataCorp, USA).

Statistical significance was defined as *p*<0.05. For multiple corrections, *p*-values were not corrected.

## Results

### Association between *MTNR1B* and gestational glucose levels

Table 2 summarizes the associations of the *MTNR1B* variants with the gestational glucose levels in the pregnant Chinese women. For FPG, rs1387153 and rs2166706 were shown to be associated with gestational FPG levels (*p*<0.05), whereas rs10830963 and rs1447352 were not found to be significantly associated with FPG levels. But interestingly, the mean level of FPG in women with each additional risk alleles for each variant was higher than those without risk alleles. For PPG, each additional risk allele increased the levels of PPG. For example, each additional G risk allele of rs10830963 significantly increased the PPG level (CC, CG and GG were found to have a mean PPG levels of 7.03 to 7.44 and 7.90 mmol/l, respectively). After adjusting for age and BMI, under the additive genetic model, each G allele of rs10830963 was found to be associated with an increased PPG level of 0.33 mmol/l (95% CI 0.23, 0.44, *p* = 8.04×10^−10^). Analyses of repeat regression after conditioning on rs10830963 revealed that each G allele was associated with an increased PPG level of 0.43 mmol/l (95% CI 0.33, 0.53, *p* = 2.59×10^−16^). Also, a risk allele of rs1387153 T, rs2166706 T and rs1447352 A was found to be significantly associated of with an increased PPG level (*p* = 5.49×10^−6^, 1.89×10^−5^ and 0.03, respectively), whereas no association was found between the *MTNR1B* variants and the other traits tested such as weight, BMI or age.

**Table 2:** Association between genetic variants of *MNTR1B* and gestational plasma glucose in pregnant Chinese woman.

*MTNR1B* Variant	Location (Chromosome11) bp	Allele frequency (Major/Minor)	genotype	FPG (mmol/l)	FPG Coefficientβ (95% CI) *p_*value^a^	PPG (mmol/l)	PPG Coefficientβ (95% CI) *p_*value^a^	Genotype Counts Normal glucose tolerance (n = 1210)	Genotype Counts Abnormal glucose tolerance (n = 775)	OR (95% CI) & *p_*value^b^	Conditioned Coefficientβ & OR (95% CI) & *p_*valuea, b on rs10830963
rs10830963	92708710	0.59/0.41	CC	4.69±0.60	0.04(−0.003, 0.09)	7.03±1.54	0.33 (0.23, 0.44) *p* = 8.04×10^−10^	475	195	1.64 (1.41, 1.90) *p* = 8.03×10^−11^	a0.43 (0.33, 0.53) *p* = 2.59×10^−16^
		(C/**G**)	CG	4.84±0.63	*p* = 0.07	7.44±1.51		569	402		b1.64(1.43, 1.87) *p* = 6.97×10^−13^
			GG	4.82±0.91		7.90±1.83		158	171		
rs2166706	92691532	0.58/0.42	CC	4.71±0.59	0.05(0.002, 0.09)	7.13±1.55	0.23 (0.13, 0.34)	442	217	1.38 (1.19, 1.59)	
		(**T**/C)	CT	4.84±0.73	*p* = 0.04	7.48±1.62	*p* = 1.89×10^−5^	550	392	*p* = 1.63×10^−5^	
			TT	4.79±0.69		7.64±1.64		187	153		
rs1387153	92673828	0.58/0.42	CC	4.71±0.59	0.05(0.002, 0.09)	7.11±1.54	0.25 (0.14, 0.36)	439	210	1.43 (1.23, 1.66)	
		(C/**T**)	CT	4.83±0.74	*p* = 0.04	7.45±1.62	*p* = 5.49×10^−6^	577	399	*p* = 1.94×10^−6^	
			TT	4.78±0.67		7.67±1.61		179	153		
rs1447352	92722761	0.69/0.31	GG	4.77±0.59	0.02(−0.03, 0.06)	7.20±1.62	0.13 (0.01, 0.24)	111	53	1.24 (1.06, 1.46)	
		(**A**/G)	AG	4.80±0.62	*p* = 0.43	7.29±1.58	*p* = 0.02	566	335	*p* = 0.007	
			AA	4.77±0.75		7.50±1.62		525	380		

Data are presented as (mean ± SD); Location information downloaded from http://asia.ensemble.org; the risk allele of the variant is showed in bold; No transformation was applied to normalize the glucose levels (Fig. S1); *p* values were not corrected for multiple comparisons.

a Calculated using multiple regression, assuming an additive model adjusted for age and BMI.

b Calculated using logistic regression, assuming an additive model adjusted for age and BMI.

### Association between *MTNR1B* variants and gestational glucose intolerance

We further analyzed the genetic association of *MTNR1B* variants for the risk of plasma glucose intolerance between the NGT and the abnormal groups including GIGT/ABN & GDM. The minor alleles of rs10830963 and rs1387153, and the major alleles of rs2166706 and rs1447352 were found to be associated with the gestational glucose intolerance under the additive genetic model (Table 2). In particular, the frequency of the risk allele G of rs10830963 was much higher in the gestational glucose intolerance group compared to that of the normal group (48.43% vs. 36.81%); adjustments for age and BMI yielded an OR of 1.64 (95% CI 1.41, 1.90) with *p* = 8.03×10^−11^. Also, the risk alleles of rs1387153 T and rs2166706 T displayed higher frequencies in the glucose intolerance pregnant women than those in the normal ones (46.26% vs. 39.12% and 60.81% vs. 54.20%, respectively). The *MTNR1B* variant rs1447352 was observed to have less influence on glucose intolerance than the other variants in this study.

### LD Structure Comparison

We compared the LD structure of these *MTNR1B* variants among the Europeans, Han Chinese in Beijing, Chinese in Metropolitan Denver and the studied pregnant population (Figure 1). The results indicated that the LD pattern in the studied women conformed to that in general populations: rs1387153 and rs2166706 were in the same LD block with each other (|D′| = 0.98, r^2^ = 0.96 in the studied women); Also, although rs10830963 and rs1447352 have high |D′| ( = 0.96 in the studied women), the 2 variants might not be in the same LD block with each other as the r^2^ was very low ( = 0.29 in the studied women); Similar to that of general population, rs10830963 was moderately linked with the LD block of rs1387153 and rs2166706 by both measures of |D′| and r^2^, but rs1447352 was not linked with the LD block by measure of r^2^ in the studied women. The relation among these 4 variants in the studied women was very close to that of Chinese in Metropolitan Denver, as the studied women and Chinese in Metropolitan Denver may mainly consist of both South and North origin Chinese people, whereas, Han Chinese in Beijing are mainly north origin.

## Discussion

This study revealed that the 4 *MTNR1B* genetic variations, which were previously reported to be associated with glucose homeostasis in general populations, were associated with the gestational postprandial glucose level and with the glucose intolerance of various degrees in the pregnant Han Chinese women. Both rs1387153 and rs2166706 were found to be tightly linked with each other and displayed the same association with gestational plasma glucose levels. The G allele of rs10830963 was shown to be the strongest signal to associate with the increased PPG level and abnormal glucose tolerance. However, rs1447352 was not found to be strongly associated with gestational glucose levels and glucose intolerance. In both general populations and our studied pregnant cohort, the rs1447352 has high values of D′ with the other 3 variants, especially with rs10830963, but the values of r^2^ between rs1447352 and these variants were lower than 0.30 which indicated that rs1447352 might not be really linked with these variants. Also, the different degrees of the association are probably related to the variant's different location in the gene. The variant rs1387153 and rs2166706 located at the >11 kb upstream of the *MTNR1B* gene, rs10830963 is intronic, whereas rs1447352 located at the ∼4.5 kb downstream of the gene (Figure 1A). Currently little knowledge about their role and regulation of *MTNR1B* gene expression is available. It is well known that gene upstream sequences and introns have various important regulatory functions, for example, intron-derived miRNAs are able to induce RNA interference. Hence it is intriguing for further research on how these different variants affect the gene function, especially in the gestational background. Based on GWAS, there are reports about that the G-allele of *MTNR1B* rs10830963 associated with defective early insulin response, beta-cell dysfunction and hepatic insulin resistance in Europeans [13], [14], and Lyssenko *et al.* have showed the pathogenic effects of MTNR1B were likely exerted via a direct inhibitory effect on beta cell [12], the particular physiological pathway of MTNR1B in glucose metabolism has not been identified, yet it is interesting that the genetic variants within the *MTNR1B* region have different influence on glucose levels during pregnancy. Considering the importance of biological rhythms for metabolic regulation and the reciprocal influence of gestational hormones, the link between the *MTNR1B* susceptibility loci and gestational glucose metabolism may reveal new mechanisms underlying glucose homeostasis during pregnancy.

In pregnant Chinese women, we observed that rs1387153 and rs2166706 were associated with gestational FPG levels, but we could not detect a significant association between the *MTNR1B* variants rs10830963, rs1447352 and the gestational FPG levels in the regression analysis. Nevertheless, we observed that for each *MTNR1B* variant with each additional risk alleles, the mean levels of FPG were higher than those without the risk alleles in the studied women. It is also noteworthy that in the current study, the effect size of FPG in pregnant women was less than that reported in the general populations. In fact, FPG levels tend to decrease during pregnancy, likely due to the effect of pregnant hormones on blood glucose and insulin resistance. Our data indicate that *MTNR1B* genetic polymorphisms could be associated with gestational FPG levels in the pregnant Chinese women.

In this study, both levels of FPG and PPG were normally distributed (Figure S1); the allelic and genotypic frequencies of all the 4 studied variants were in Hardy-Weinberg equilibrium (Table S1); and the result of LD tests in these pregnant women was concordant with the LD pattern in the general population [20]. Since the studied sample population of pregnant women was limited to Chengdu in China, it will be necessary to confirm our findings through studies of populations of other districts and ethnicities. Interestingly, *MTNR1B* rs10830963 and rs1387153 were reported to be linked to the risk of GDM in Korean women [28], as well, rs10830963 was recently found to be associated with beta-cell function in a Chinese pregnant Population [29], although both studies did not test the association of the *MTNR1B* variants with different gestational glucose levels and glucose intolerance.

In summary, our data suggests that the genetic variants within *MTNR1B* gene are associated with gestational glucose levels and gestational glucose intolerance. To our knowledge, this is the first report on the association in a cohort of pregnant Chinese women. This work extended the research on MTNR1B from the general population into the context of pregnancy and provided new information on associations of *MTNR1B* polymorphisms in the Chinese population.

## Supporting Information

Figure S1 **Histogram of fasting and 1 hour postprandial glucose levels.** Both levels of FG & 1 h PPG (fg & p1hrs) in the 1,985 pregnant Chinese women at 24–28 gestational weeks were normally distributed.
(TIF)

Table S1 Quality control results of the variants and the p-value for Hardy-Weinberg Equilibrium.
(DOC)

Flowchart S1 Screening for gestational glucose intolerance in pregnant Chinese women. Briefly, a stepwise approach for all pregnant women was used. Step 1 is a screening 50 g, 1 h glucose challenge test, both the fasting glucose and the 1 hour postprandial glucose levels were determined. Women with FPG ≥5.8 mmol/l or 1 h PPG ≥7.8 mmol/l were defined as a positive GCT result and would be suggested to go to step 2. Step 2 is a 100 g, 3 h OGTT. NDDG cut-off for diagnosis GDM was employed. The high-risk pregnant women with demonstrable risk factors might be suggested to undergo a random blood glucose test at the first antenatal visit. If they had FPG ≥7.0 mmol/l or a random PPG ≥11.1 mmol/l, and this result was confirmed on a subsequent day, the women would be diagnosed with GDM or DM (T1DM and previous T2DM with elevated HgA1C prior to 20 gestational weeks) without diagnostic OGTT. There were 1992 pregnant women who agreed to join the study. 196 women with high risk factors of diabetes, together with 1779 women underwent 50 g GCT. Among these women, 1209 women were negative in GCT; only 1 woman was later rescreened for 100 g OGTT due to suspected macrosomia. The other 766 women were positive in GCT, 4 of them were confirmed with fg>7 mmol/l or pg>11 mmol/l. Although the rest 762 women with positive GCT were suggested to undergo 100 g OGTT, 654 women actually underwent 100 g OGTT. Among these 654 women, 349 women were positive in 100 g OGTT result. In detail, 72 of these 349 women were GDM according to NDDG diabetes criteria, 5 were GIGT and 113 were just abnormal in one glucose level without reaching the thresholds. For the other women with high risk factors of diabetes, 7 women (T1DM and T2DM) were excluded from the current study, and 9 women directly underwent 100 g OGTT, 2 of these 9 women were normal in 100 g OGTT result.
(DOC)
