# Supplementary figures and images for: Association of Genetic Variants of Melatonin Receptor 1B with Gestational Plasma Glucose Level and Risk of Glucose Intolerance in Pregnant Chinese Women
Source: PLoS One. 2012 Jul 2;7(7):e40113. doi: 10.1371/journal.pone.0040113 (PMC3388040; doi:10.1371/journal.pone.0040113)

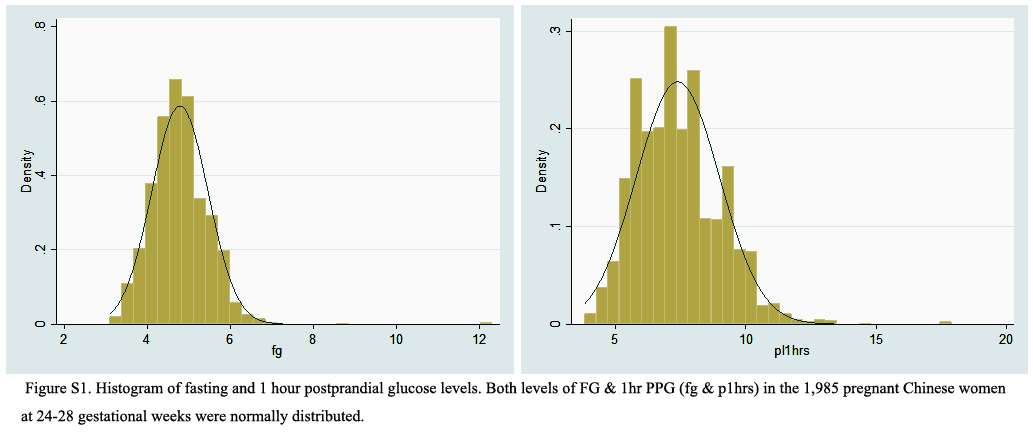

Supplement: Figure S1 — Histogram of fasting and 1 hour postprandial glucose levels. Both levels of FG & 1 h PPG (fg & p1hrs) in the 1,985 pregnant Chinese women at 24–28 gestational weeks were normally distributed. (TIF) [file pone.0040113.s001.tif]
