# Supplementary material for: Association of Genetic Variants of Melatonin Receptor 1B with Gestational Plasma Glucose Level and Risk of Glucose Intolerance in Pregnant Chinese Women
Source: PLoS One. 2012 Jul 2;7(7):e40113. doi: 10.1371/journal.pone.0040113 (PMC3388040; doi:10.1371/journal.pone.0040113)
Supplement: Table S1 — Quality control results of the variants and the p-value for Hardy-Weinberg Equilibrium. (DOC) [file pone.0040113.s002.doc]

**Table S1.** Quality control results of the variants and the p-value for Hardy-Weinberg Equilibrium (HWE)

| **Gene** | **Variant** | **Call success rate** | **Concordance rate** | ***p*-value for HWE** |
| --- | --- | --- | --- | --- |
| *MTNR1B* | rs10830963 | 99.24% | 100% | 0.47 |
|  | rs2166706 | 98.76% | 99% | 0.91 |
|  | rs1387153 | 98.59% | 100% | 0.28 |
|  | rs1447352 | 98.66% | 99% | 0.13 |
