# Supplementary material for: Association of Genetic Variants of Melatonin Receptor 1B with Gestational Plasma Glucose Level and Risk of Glucose Intolerance in Pregnant Chinese Women
Source: PLoS One. 2012 Jul 2;7(7):e40113. doi: 10.1371/journal.pone.0040113 (PMC3388040; doi:10.1371/journal.pone.0040113)
Supplement: Flowchart S1 — Screening for gestational glucose intolerance in pregnant Chinese women. Briefly, a stepwise approach for all pregnant women was used. Step 1 is a screening 50 g, 1 h glucose challenge test, both the fasting glucose and the 1 hour postprandial glucose levels were determined. Women with FPG ≥5.8 mmol/l or 1 h PPG ≥7.8 mmol/l were defined as a positive GCT result and would be suggested to go to step 2. Step 2 is a 100 g, 3 h OGTT. NDDG cut-off for diagnosis GDM was employed. The high-risk pregnant women with demonstrable risk factors might be suggested to undergo a random blood glucose test at the first antenatal visit. If they had FPG ≥7.0 mmol/l or a random PPG ≥11.1 mmol/l, and this result was confirmed on a subsequent day, the women would be diagnosed with GDM or DM (T1DM and previous T2DM with elevated HgA1C prior to 20 gestational weeks) without diagnostic OGTT. There were 1992 pregnant women who agreed to join the study. 196 women with high risk factors of diabetes, together with 1779 women underwent 50 g GCT. Among these women, 1209 women were negative in GCT; only 1 woman was later rescreened for 100 g OGTT due to suspected macrosomia. The other 766 women were positive in GCT, 4 of them were confirmed with fg>7 mmol/l or pg>11 mmol/l. Although the rest 762 women with positive GCT were suggested to undergo 100 g OGTT, 654 women actually underwent 100 g OGTT. Among these 654 women, 349 women were positive in 100 g OGTT result. In detail, 72 of these 349 women were GDM according to NDDG diabetes criteria, 5 were GIGT and 113 were just abnormal in one glucose level without reaching the thresholds. For the other women with high risk factors of diabetes, 7 women (T1DM and T2DM) were excluded from the current study, and 9 women directly underwent 100 g OGTT, 2 of these 9 women were normal in 100 g OGTT result. (DOC) [file pone.0040113.s003.doc]

**Flowchart S1 Screening for gestational glucose intolerance in pregnant Chinese women**

Briefly, a stepwise approach for all pregnant women was used:.

Step 1 is a screening 50g, 1h glucose challenge test, both the fasting glucose and the 1 hour postprandial glucose levels were determined. Women with FPG ≥ 5.8mmol/l or 1h PPG ≥ 7.8mmol/l were defined as a positive GCT result and would be suggested to go to step 2.

Step 2 is a 100g, 3h OGTT. NDDG cut-off for diagnosis GDM was employed.

The high-risk pregnant women with demonstrable risk factors might be suggested to undergo a random blood glucose test at the first antenatal visit. If they had FPG ≥ 7.0mmol/l or a random PPG ≥ 11.1mmol/l, and this result was confirmed on a subsequent day, the women would be diagnosed with GDM or DM (T1DM and previous T2DM with elevated HgA1C prior to 20 gestational weeks) without diagnostic OGTT.

There were 1992 pregnant women who agreed to join the study. 196 women with high risk factors of diabetes, together with 1779 women underwent 50g GCT. Among these women, 1209 women were negative in GCT; only 1 woman was later rescreened for 100g OGTT due to suspected macrosomia. The other 766 women were positive in GCT, 4 of them were confirmed with fg>7 mmol/l or pg>11mmol/l. Although the rest 762 women with positive GCT were suggested to undergo 100g OGTT, 654 women actually underwent 100g OGTT. Among these 654 women, 349 women were positive in 100g OGTT result. In detail, 72 of these 349 women were GDM according to NDDG diabetes criteria, 98 were GIGT and 185 were just abnormal in one glucose level without reaching the thresholds. For the other women with high risk factors of diabetes, 7 women (T1DM and T2DM) were excluded from the current study, and 9 women directly underwent 100g OGTT, 2 of these 9 women were normal in 100g OGTT result.
